# SARS-CoV-2 replicates in the placenta after maternal infection during pregnancy

**DOI:** 10.3389/fmed.2024.1439181

**Published:** 2024-09-04

**Authors:** Anda-Petronela Radan, Patricia Renz, Luigi Raio, Anna-Sophie Villiger, Valérie Haesler, Mafalda Trippel, Daniel Surbek

**Affiliations:** ^1^Department of Obstetrics and Feto-Maternal Medicine, University Hospital of Bern, University of Bern, Bern, Switzerland; ^2^Department for Biomedical Research, University of Bern, Bern, Switzerland; ^3^Department of Pathology, University of Bern, Bern, Switzerland

**Keywords:** COVID-19, placenta, SARS-CoV-2, SARS-CoV-2 replication, stillbirth

## Abstract

**Objectives:**

Pregnant women are at increased risk for severe SARS-CoV-2 infection and adverse neonatal outcome, primarily preterm birth and stillbirth. Our study aimed to investigate to which extent SARS-CoV-2 affects placental tissue and if viral replication within the placenta is evident, thus if there is a correlation between placental damage and adverse pregnancy outcome such as stillbirth.

**Methods:**

We prospectively collected placentas from 61 SARS-CoV-2 infected pregnant women and 10 controls. Histopathological, immunohistochemical, and *in situ* hybridization studies were performed on all placentas with antibodies for SARS-CoV-2 proteins, ACE2, various immune cells, and inflammatory markers or probes for SARS-CoV-2 genes and an antisense strand.

**Results:**

The measured scores of SARS-CoV-2 glycoprotein, nucleocapsid, and antisense strand indicating replication correlated with both the severity of maternal symptoms and presence of stillbirth. Specifically, 15/61 placentas exhibited replication, while the three cases with stillbirth had high or maximal replication scores. ACE2-H-score was significantly higher in COVID-19 patients, while the expression of various immune cells did not differ statistically. In multivariate analysis, presence of maternal comorbidities correlated with presence of severe COVID-19 infection.

**Conclusion:**

We report evidence of active *in vivo* SARS-CoV-2 replication in the placenta after maternal infection in pregnancy in a case–control setting in a large population. Intensity of placental viral replication as well as viral levels were higher in women with severe or critical COVID-19 disease, supporting the rationale that severity of maternal SARS-CoV-2 infection could correlate with the severity of placentitis. Replication was maximal in cases of stillbirth, which suggests direct placental involvement in the pathophysiology of this dramatic outcome. Continuing to advocate for preventive measures against COVID-19 during pregnancy, including (re)vaccination, as well as appropriately counseling women with diagnosed infection, are of utter importance.

## Introduction

Pregnancy increases the risk for a severe course of COVID-19 disease (1). Whereas the pathophysiology of maternal complications is well described, the mechanisms leading to poor fetal outcome remain poorly understood (2, 3).

Several publications noted evidence of the triad chronic histiocytic intervillositis (CHI), massive perivillous fibrin deposition (MPFD), and trophoblast necrosis (TN) in association with COVID-19 miscarriage and stillbirth, in some cases being present in completely asymptomatic women (4–13). These lesions, especially if occurring together (CHI, MPFD, and TN), appear to be typical for SARS-CoV-2 and can represent the underlying cause for acute placental insufficiency and fetal demise, yet as separate entities they can also be part of an alloimmune maternal response of different etiology (5–13).

Given the inconsistency of histopathological data, a variety of investigations have been previously carried out by various research groups, leading to a paucity of publications, generally small case series or dispersed case reports, with often conflicting results. For example, whereas one study reported 100% presence of SARS-CoV-2 spike glycoprotein in the analyzed placentas, others report either no evidence of viral RNA in their cohorts, or in a significant smaller number of cases (14–17).

Furthermore, there is a great difference in severity of placental infection and pregnancy outcome related to the various serotypes. For example, the ancestral and the Omicron serotypes caused less placentitis and less intrauterine deaths, as opposed to delta, as shown in a recent registry study (18). This could partially explain the inconsistency of results originating from small cohorts. In December 2021, a panel of experts released the first standardized definition of placental infection with SARS-CoV-2, in an attempt to minimize misclassification. According to these, confirmation of definitive placental infection requires evidence of active viral replication in placental tissue, whereas the sole evidence of viral RNA or protein in placental tissue can only provide evidence of probable transmission (19). Furthermore, according to consensus, evidence of replication should always include appropriate negative controls (3).

*In situ* hybridization (ISH) and/or placental RT-PCR are recommended for diagnosis of transplacental transmission, as these methods appear to be superior to placental swab PCR testing, immunohistochemistry (IHC), and electronic microscopy (EM) for this purpose (19, 20).

These insights underline the necessity for large, well-conducted case–control studies to address the extent of placental SARS-CoV-2 infection and its impact on pregnancy outcomes, by following current standards of investigation and scientific reporting.

We hypothesized that SARS-CoV-2 replicates in the placenta of infected mothers. We aimed to conduct a prospective study in a SARS-CoV-2 infected pregnant population and to perform histopathological analysis, PCR-testing, IHC and ISH at placental level, and assess the extent of damage, as well as vertical transmission and the immune response at the maternal-fetal interface, compared to corresponding controls. Furthermore, we intended to investigate if SARS-CoV-2 viral replication within the placenta is evident, and if there is a correlation between placental damage and adverse pregnancy outcome such as stillbirth.

## Materials and methods

We performed a nested case–control study, including 61 cases and 10 controls in our analysis. Cases included all women diagnosed with SARS-CoV-2 infection during pregnancy, irrespective of disease severity or gestational age at infection, who were managed at our tertiary referral hospital between May 2020 to October 2022, and who provided written participation consent.

Controls were selected from a population without SARS-CoV-2 infection during pregnancy in the predefined study period. Selection occurred based on gestational age at delivery and comorbidities, which could have influenced outcomes in a similar manner to SARS-CoV-2. We chose a nested case–control design in order to reduce costs and efforts of data collection and analysis.

Several cases were highlighted. For a simplified presentation, these were annotated with the symbol “i” for cases (i = infected) and “c” for controls (c = control).

Data were collected prospectively. Diagnosis of infection was made by SARS-CoV-2-PCR in a nasopharyngeal swab.

Beta B.1.351 variant was dominant in Switzerland until the beginning of May 2021, followed by Gamma P.1 for a period of 2 weeks, when Delta B.1.617.2 took over and continued to be dominant until mid-December 2021. Ever since, Omicron B.1.1.529 was the dominant variant in our country.

Serotype analysis was only conducted in one single placenta, more specific in Case 2i, where stillbirth occurred at 30 weeks of gestation. This case occurred at the very beginning of the Omicron outbreak, and serotype was analyzed as an exception in this case, because little was known about the new variant (i.e., Omicron), and the treating clinicians suspected high virulence. However, the affected case was ultimately infected with Delta.

The Cantonal Ethics Committee in Bern has approved conduction the study (registration-number BASEC 2020-02610). No external funding was received for the study.

### Tissue preparation

A piece of tissue collected from the maternal side of the placenta was fixed with 4% paraformaldehyde for 30–36 h (h) at 4°Celsius (C) immediately after birth. It was then embedded in paraffin, and sections were cut into 6 μM pieces using the HM340E-microtome.

### Immunohistochemistry

Placental sections were deparaffinized and rehydrated, then blocked with 10% goat serum, and 1% bovine serum albumin (BSA) in TBS for 1 h at room temperature. Subsequently, sections were incubated overnight at 4°C with following primary antibodies: CD3/CD8/CD68/CD86,/CD11b,/TNFa/ACE2,/SARS-CoV-2-nucleocapsid and SARS-CoV-2-spike-glycoprotein. After washing, sections were blocked with peroxidase for 15 min and subsequently incubated with peroxidase-labeled secondary antibody followed by diaminobenzamidine.

### *In situ* hybridization

RNAscope® fluorescent *in situ* hybridization was conducted on formalin-fixed, paraffin-embedded placentas using the RNAscope® 2-Plex Detection Kit (Chromogenic) according to the manufacturer’s protocol for SARS-CoV-2 Antisense strand of the orf1ab gene, SARS-CoV-2 S gene encoding the spike protein and IL6.

### Image analysis

ACE2 staining was quantified for immunoreactivity using Qupath. Histological (H) score calculated by multiplying the percentage of stained placental tissue (decidua and/or villi) by the average staining intensity (between 0 and 3). The program outputs the total number of detected cells, which is used to calculate the H-score. H-scores can vary from 0 (no expression) to 300 (intensity of staining 3 in 100% of the cells analyzed).

For CD3/CD8/CD68/CD86/TNFa/CD11b-staining, hotspot areas were identified.

The method of the immunoreactive score (IRS; 0–1 = negative, 2–3 = mild, 3–8 = moderate, 9–12 = strongly positive) was used for all SARS-CoV-2 and IL6 staining by multiplying the percentage of positive cells (0 = no positive cells, 1 = <10%, 2 = 10–50%, 3 = 51–80%, 4 = >80% positive cells, respectively) and the staining intensity (0 = no color reaction, 1 = mild, 2 = moderate, 3 = intense reaction, respectively).

The researchers analyzing the placentas were blinded to the group. Data were analyzed by at least two persons in every case.

### Statistical analysis

Mean values and standard deviation (SD) were calculated for continuous variables and percentages for qualitative variables. A student *t*-test and Fisher’s exact test were used for continuous parametric variables and binary variables, respectively. Multiple linear regression was performed to analyze risk factors for presence of severe or critical COVID-19 illness, respectively risk factors for presence of viral replication in the placenta. Logistic regression analysis was performed to identify if the time point of infection was associated with presence of severe COVID-19 disease. Significance was set at a *p* value of <0·05. Statistical analysis was carried out with RStudio for Windows.

## Results

Altogether, 41/71 (57.74%) cases were recruited in the pre-Delta period, 18/71 (25.35%) during Delta and 11/71 (15.49%) during Omicron dominance. As mentioned before, serotype analysis was only conducted in one single placenta, more specific in Case 2i.

88.5% (54/61) of the cases suffered from asymptomatic, mild/moderate SARS-CoV-2 infection, according to the National Institutes of Health (NIH) criteria for severity of the disease (20). 11·47% (7/61) presented with severe or critical illness and were managed as inpatient. 9.83% (6/61) required intensive care unit (ICU) admission and ventilation and consequently underwent emergency delivery.

4/41 (9.75%) cases recruited in the pre-Delta period presented with severe or critical illness, versus 3/18 (16.66%) cases during Delta dominance, versus no case during the Omicron phase. The difference between the rate of severe or critical cases between the pre-Delta and Delta periods was not statistically significant (*p* = 0.44, chi-squared test).

No maternal deaths were recorded. In two women with severe/critical illness (28.57%, 2/7), infection occurred in the second trimester of pregnancy (median [range] gestational age = 18 [17–19]). In the remaining 5/7 (71.42%), infection occurred in the third trimester of pregnancy (median [range] gestational age = 30 [29–32]). Further patient characteristics and outcomes are depicted in Table 1.

**Table 1:** Patient characteristics and maternal and fetal outcome.

Characteristics	Cases	Controls	*p*
Maternal age (mean, ±SD)	30.44 ± 4.38	33.30 ± 3.20	0.0523^*^
Presence of GDM (*n*/*N*)	14/61 (22.95%)	3/10 (30%)	0.6490^**^
BMI^*^ (kg/m^2^) (mean, ±SD)	26.82 ± 6.32	28.93 ± 4.67	0.3165^*^
Multiparity (*n*/*N*)	26/61 (43.33%)	2/10 (20%)	0.1748^**^
Comorbidities^***^ (*n*/*N*)	13/61 (21.13%)	4/10 (40%)	0.1993^**^
**Outcome**	**Cases**	**Controls**	***p***
Gestational age at delivery (days) (mean, SD)	257 ± 43.56	250.80 ± 51.47	0.6854^*^
Preterm delivery (*n*/*N*)	17/61 (27.87%)	3/10 (30%)	0.8896^**^
Cesarean delivery (*n*/*N*)	27/61 (45%)	4/10 (40%)	0.8011^**^
Birth weight^#^ (mean, ±SD)	2918.67 ± 928.03	2414.5 ± 1210.20	0.1320^*^
IUGR/SGA (*n*/*N*)	8/61 (13.11%)	5/10 (50%)	0.0051^**^
Neonatal arterial pH (mean, SD)	7.13 ± 1.002	7.17 ± 0.1095	0.000001^*^
Neonatal APGAR (mean, SD)	8.69 ± 1.55	7.67 ± 1.80	0.9004^*^
Neonatal admission at ICU (*n*/*N*)	6/61 (9.84%)	2/10 (20%)	0.0634^*^
Misscarriage/stillbirth (*n*/*N*)	3/61 (4.92%)	1/10 (10%)	0.5182^*^

BMI, Body mass index; GDM, Gestational diabetes; ICU, Intensive care unit; IUGR, Intrauterine growth restriction; SD, Standard deviation; SGA, Small for gestational age; ^*^Student *t* test; ^**^chi^2^ test; ^***^Comorbidities: arterial hypertension, asthma, juvenile arthritis, wide complex tachycardia, Hashimoto thyroiditis, psychiatric condition; ^#^Birth weight was not adjusted for gestational age.

**Figure 1:**
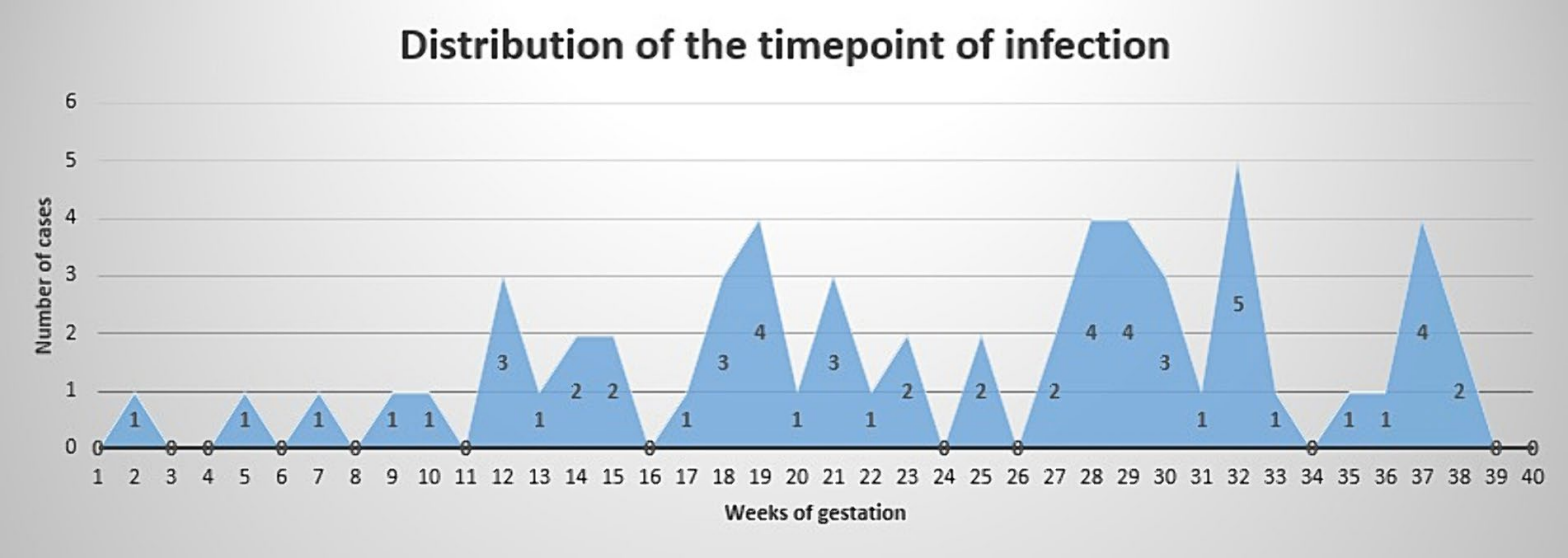
Time point of infection. *X* axis shows weeks of gestation. *Y* axis shows number of cases.

Time point of infection is depicted in Figure 1. Logistic regression showed no correlation between time point of infection and presence of severe/critical illness (*p* = 0.4497).

**Figure 2:**
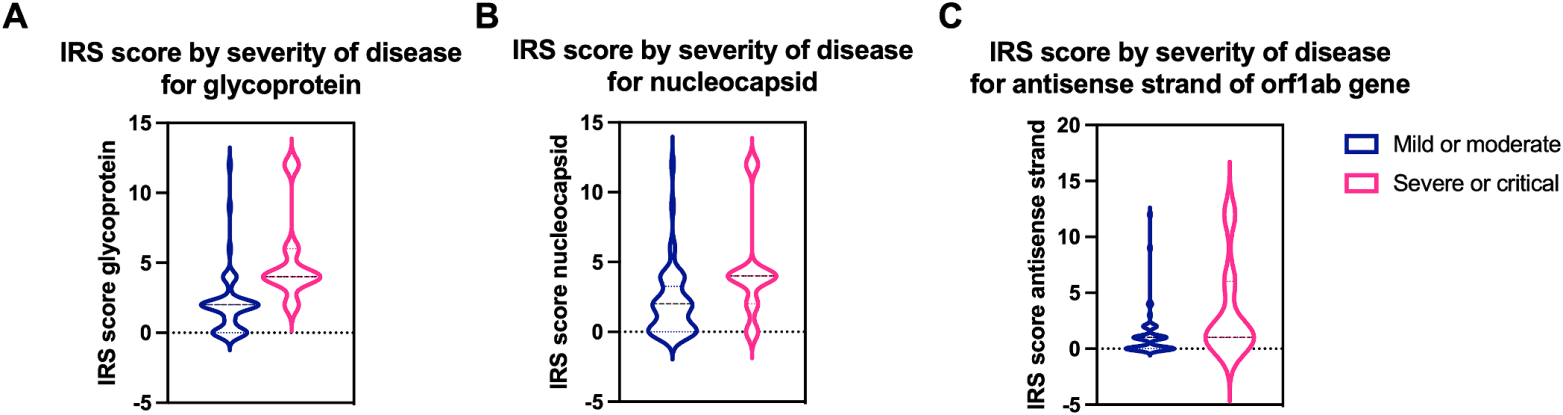
Quantification of SARS-CoV-2 specific proreins and virus replication in mild/moderate and severe/critical COVID-19 cases. IRS score for **(A)** the spike glycoprotein (*p* = 0.0013) and **(B)** the nucleocapsid protein (*p* = 0.03) is significantly higher in severe or critical cases (pink) compared to mild or moderate cases (blue). **(C)** A significantly higher IRS score (*p* = 0.034) for the virus replication was detected in severe or critical cases. Data are represented as mean ± SD.

ACE2 H-score was significantly higher in cases versus controls (Figure 2B). If analyzed separately in the decidua, mean ACE2 H-score was 60.23 (±SD 44.02) in cases versus 25.06 (±SD 11.84) in controls (*p* = 0.0149) (Figure 2). Reversely, ACE2 H-Score in the villi was not significantly different between cases and controls (*p* ≥ 0.05). ACE2 H-score was significantly higher if infection occurred in the first half of pregnancy (*p* = 0.0052).

**Figure 3:**
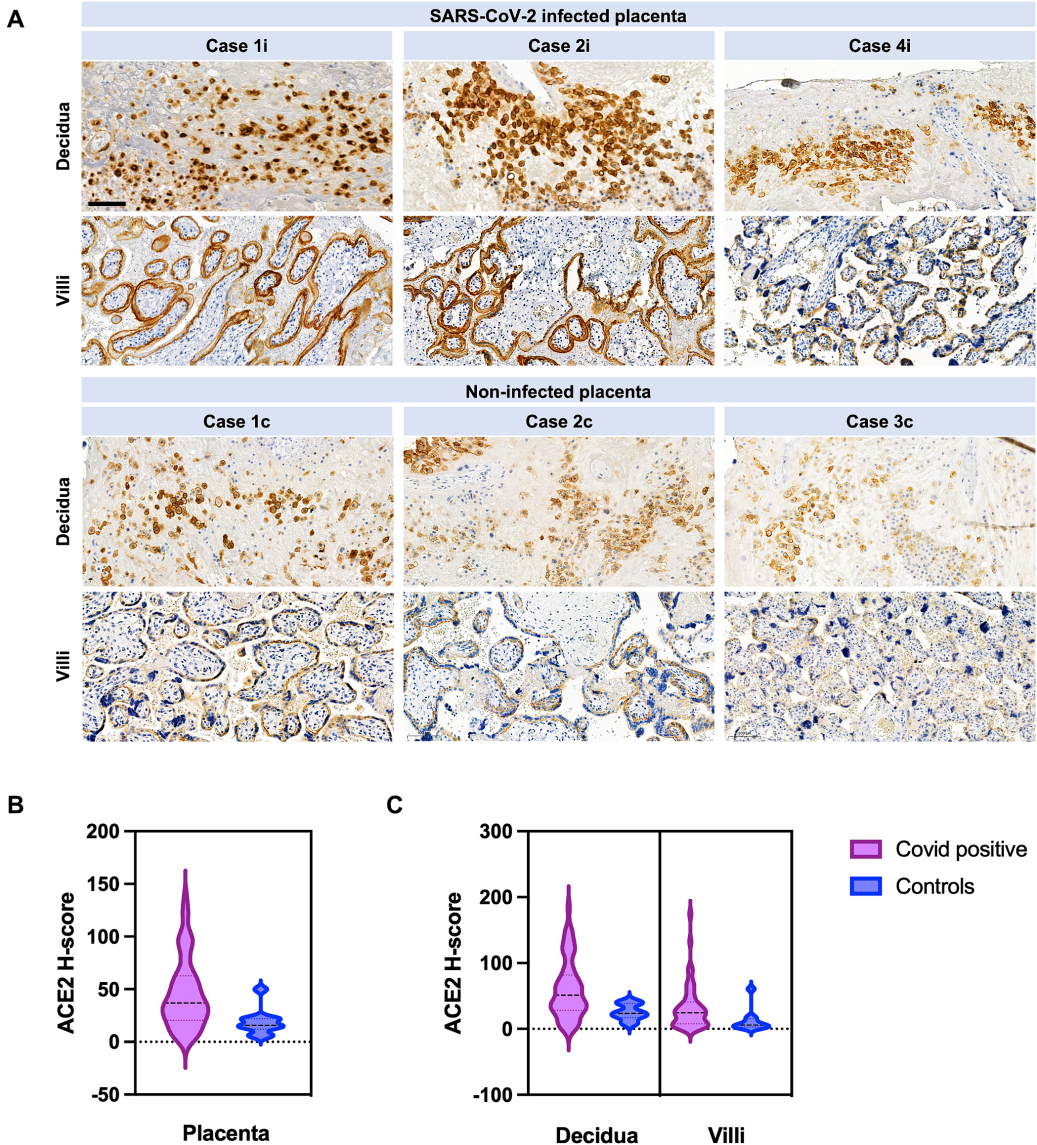
ACE2 in SARS-CoV-2 infected versus not infected placentas, with distribution between villi and decidua. **(A)** Case 1i images show COVID-19 miscarriage at 18 weeks of gestation. Case 2i images represent COVID-19 stillbirth at 30 weeks of gestation. Case 4i are images of a COVID-19 patient with severe course of the disease (pneumonia with decompensation) who was infected at 32 weeks of gestation and delivered at 33 1/7. The control cases images are placentas from COVID-19 negative women. Case 1c (control) are images of a placenta after stillbirth at 20 weeks, case 2c (control) images are from a term birth, and case 3c (control) was an IUGR case. Scale bar: 100 μm. **(B)** ACE2 H-Score quantification in the entire placenta. ACE2 H-score was significantly increased in COVID-19 cases (violet) compared to control cases (blue) (*p* = 0.0134). **(C)** ACE2 H-score was significantly increased in COVID-19 cases (violet) in the decidua compared to controls (blue) (*p* = 0.0149). No significant difference was observed in villi between COVID-19 (violet) and control cases (blue) (*p* = 0.07). Data are represented as mean ± SD.

Expression of CD3/CD8/CD11b/CD68/CD86 was not statistically different between cases and controls (Table 2; Figures 3A–H). Furthermore, expression of TNFa and IRS for IL6 were not significantly different between the two groups (*p* = 0.22 and *p* = 0.62, respectively) (Figures 3I,J).

**Table 2:** Distribution of inflammatory markers in the maternal and fetal side of the placenta.

Type of inflammatory marker	Localization	Hotspots (mean, ±SD)	Hotspots (mean, ±SD)	*p* (*t* student test)
		COVID positive	Controls	
CD3	Decidua	52.70 (±35.75)	50.3 (±39.88)	0.846
	Villi	17.28 (±32.21)	14.75 (±19.77)	0.811
CD8	Decidua	38.61 (±31.06)	32.7 (±20.51)	0.564
	Villi	22.76 (±47.46)	18.45 (±27.72)	0.781
CD11b	Decidua	63.11 (±98.32)	21.3 (±22.4)	0.187
	Villi	43.88 (±89.32)	13.6 (±20.83)	0.292
CD68	Decidua	3.85 (±2.06)	2.46 (±2.54)	0.060
	Villi	2.97 (±1.46)	2.26 (±1.73)	0.169
CD86	Villi	14.95 (±4.75)	12.95 (±2.96)	0.203

An overview of the histopathological changes in the placentas is depicted in Table 3.

**Table 3:** Placental histopathology in SARS-CoV-2 positive cases versus controls.

Outcomes	SARS-CoV-2 positive cases	Controls	*p* (chi^2^-test)
Placental weight < 10th centile	24/61 (39.34%)	4/10 (40%)	0.969
Signs of maternal malperfusion			
Infarction	8/61 (13.11%)	2/10 (20%)	0.562
Increased perivillous fibrin deposition	2/61 (3.27%)	0/10	
Accelerated villous maturation	3/61 (4.91%)	1/10 (10%)	0.518
Tenney-Parker change	2/61 (3.11%)	0/10	
Decidual vasculopathy	0/61	0/10	
Increase of intervillous fibrin	3/61 (4.91%)	0/10	
Retroplacental hemorrhage	2/61 (3.27%)	0/10	
Intervillous thrombosis	4/61 (6.55%)	0/10	
Signs of fetal malperfusion			
Thrombi in the fetal circulation	3/61 (4.91%)	0/10	
Avascular villi	1/61 (1.63%)	0/10	
Karyorrhexis	1/61 (1.63%)	0/10	
Delayed villous maturation	0/61	0/10	
Chorangiosis	0/61	0/10	
Inflammatory changes			
Chorioamnionitis	7/61 (11.47%)	1/10 (10%)	0.891
Chronic histiocytic intervillositis	1/61 (1.63%)	0/10	
Chronic villitis	3/61 (4.91%)	0/10	
Chronic deciduitis	0/61	0/10	
Subchorionitis	0/61	0/10	
Choriovasculitis	3/61 (4.91%)	2/10 (20%)	0.084
Fetal vasculitis	2/61 (3.27%)	1/10	
Other			
Placenta accreta spectrum	0/61	0/10	
Marginal insertion of the umbilical cord	8/61 (13.11%)	0/10	
Hypercoiling of the umbilical cord	0/61	0/10	
Phagocytosis of meconium	3/61 (4.91%)	1/10 (10%)	0.0518
Diffuse villous oedema	0/61 (0%)	0/10	

**Figure 4:**
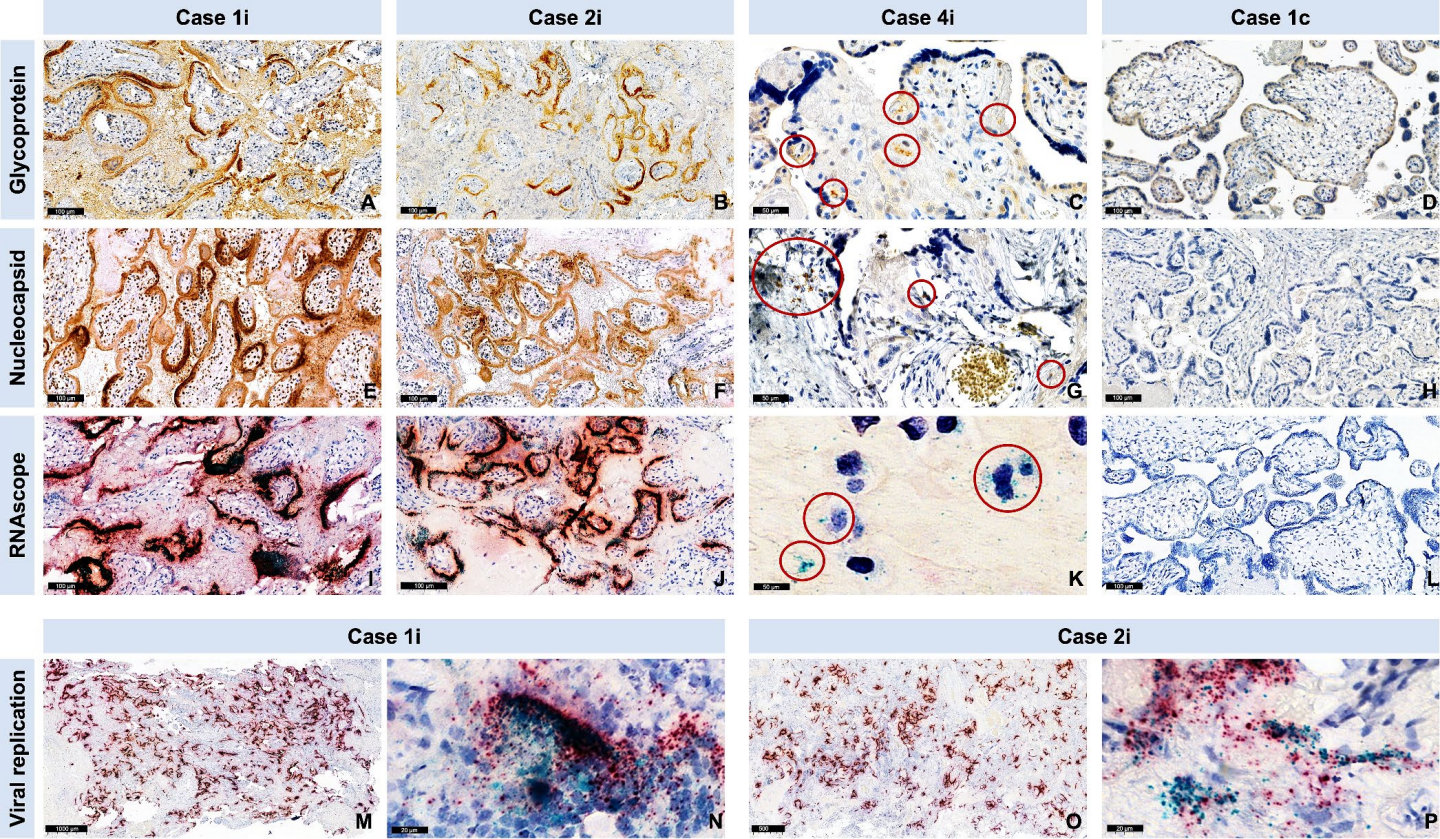
Evidence of SARS-CoV-2 nucleocapsid and glycoprotein in SARS-CoV-2 infected placentas versus controls, as well as evidence of viral replication with RNAscope®. Cases are identical to Figure 3. **(A–D)** Representative images for the spike glycoprotein and **(E–H)** the nucleocapsid protein staining of cases and a control (negative). The red circles in case 3 point to a positive signal for spike glycoprotein **(C)**, nucleocapsid protein **(G)**. **(I–P)** Representative images of the RNAscope with magenta for SARS-CoV-2 S gene encoding the spike protein and cyan for SARS-CoV-2 Antisense strand of the orf1ab gene (virus replication). **(K)** Red circles point to a positive signal for virus replication. **(M)** Representative overview and **(N)** representative high magnification image after RNAscope staining of case 1i show high viral replication (cyan). **(O)** Representative overview and **(P)** representative high magnification image after RNAscope staining of case 2i show high viral replication (cyan).

**Figure 5:**
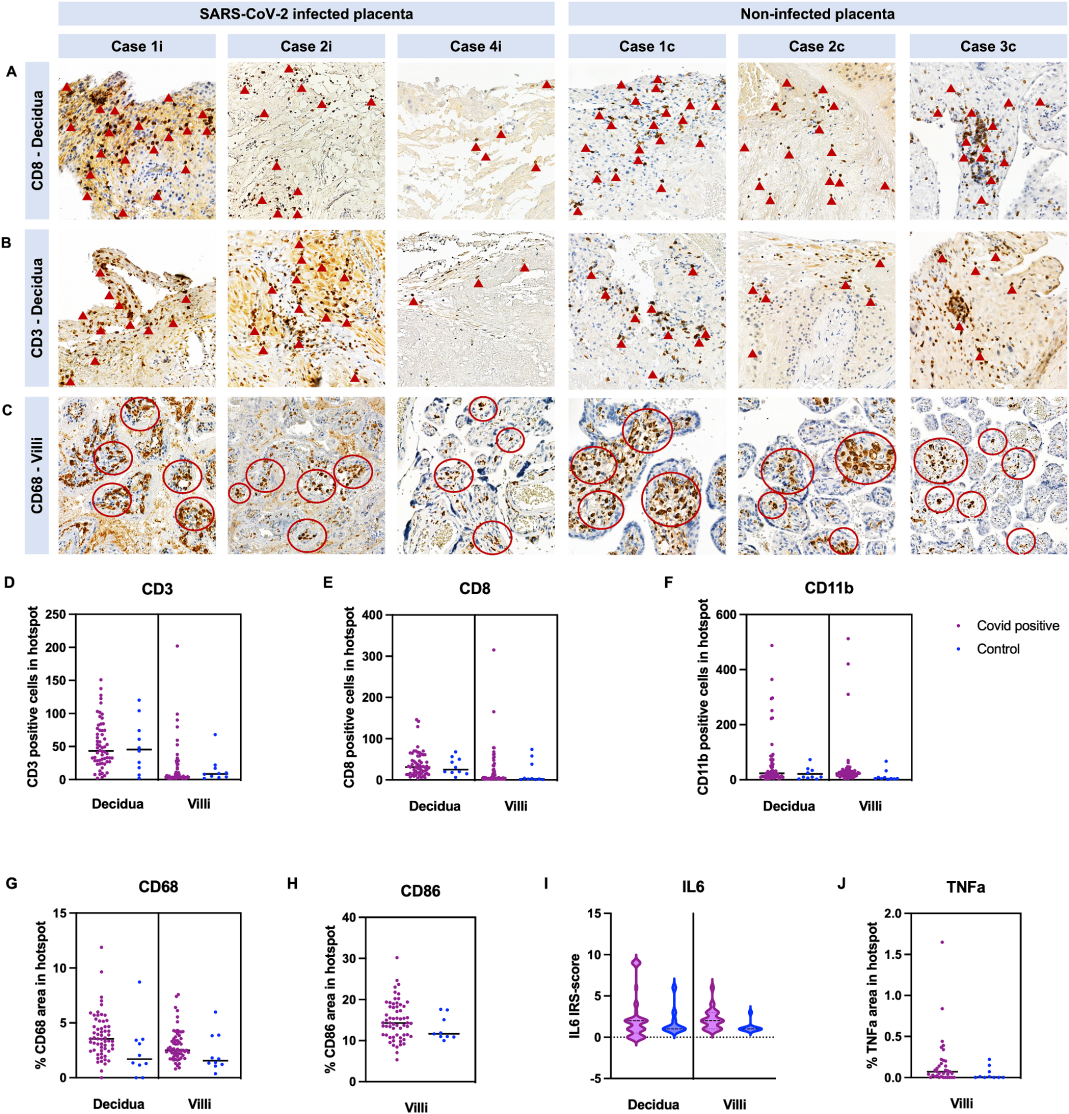
Quantification of immune cells in COVID-19 and control placentas. Cases are identical to Figure 3. Representative images of COVID-19 cases and images of control cases with **(A)** CD8+ staining in the decidua, with **(B)** CD3+ staining in the decidua and with **(C)** CD68+ staining in the villous stroma. **(A,B)** Arrowheads point to some positive CD3 and CD8 cells. **(C)** Red circles indicate hotspots of CD68 positive cells. **(D–F)** Quantification of CD3-, CD8-, and CD11b positive cell number in hotspots in the decidua and villi. **(G,H)** Quantification of positive area of CD68 and CD86 staining in hotspots in the decidua and villi. **(I)** IRS-score of *IL6* and **(J)** positive area of TNFa were additionally analyzed. In all cases, no significant difference was detected between COVID-19 and control cases (see Table 2, data represented as mean ± SD).

In the three cases with stillbirth/miscarriage, ISH showed intense viral replication at placental level. IRS was maximal (IRS = 12) for the antisense strand of the orf1ab gene in two cases (Case1i and Case2i, miscarriage at 18 and stillbirth at 30 weeks of gestation, respectively), and strongly positive (IRS = 9) for a Case3i (miscarriage at 18 weeks of gestation). Furthermore, IRS’s for SARS-CoV-2 glycoprotein (IHC), nucleocapsid (IHC), and RNA (S gene encoding for spike protein) (ISH) were also maximal in Case1i and Case2i (IRS = 12), and strongly positive (IRS = 9) in Case3i, demonstrating detection of the virus at both protein and RNA levels (Figures 4, 5). In a control where stillbirth occurred, Case1c (stillbirth at 21 weeks of gestation, no SARS-CoV-2 infection), IRS was 0 for all examinations. Mild to moderate IRS’s (IRS = 2–6) for the antisense strand of the orf1ab gene, demonstrating replication of lower intensity, were noted in further 12 cases, leading to a total of 15 placentas with evidence of viral replication (15/61, 24.59%). On a deeper analysis, IRS for glycoprotein (IHC), nucleocapsid (IHC), and for the antisense strand of the orf1ab gene (ISH) was significantly higher in placentas originating from patients with severe/critical disease (*p* = 0.0013, *p* = 0.03, and *p* = 0.034, respectively) (Figure 4). In all cases with evidence of viral replication, IHC was positive for viral proteins (Figure 5).

All cases with presence of intense replication (3/61, 4.91%) occurred during the Delta dominance.

Mean duration of time (weeks) from diagnosis of infection to delivery (thus placenta staining) was significantly lower in patients with versus without evidence of replication [8.31 versus 15.58 (weeks), *p* = 0.0189].

In all controls, IRS for the antisense strand of the orf1ab gene was 0.

We performed multiple linear regression to analyze the risk factors for presence of severe/critical illness, by considering maternal age, presence of comorbidities (as described in Table 1), gestational diabetes and adiposity (body mass index >25 kg/m^2^). Only presence of comorbidities was significantly associated with severe/critical illness (*p* = 0.0193). Further, we performed multiple linear regression to analyze if any of the above were risk factors for viral replication in the placenta, and found no association (*p* ≥ 0.05 for all). Further obstetrical outcomes are depicted in Table 4.

**Table 4:** Fetal outcomes in the cases with and without viral replication.

	Cases with evidence of viral replication (stillbirth excluded)	Cases without viral replication	*p*
Gestational age at delivery(days)	255.08 (± 21.77)	269.42 (±17.79)	0.0215
Preterm delivery (*n*/*N*, %)	7/12 (58.33%)	7/46 (15.21%)	0.0018
Birth weight^#^	2628.46 (± 882.64)	3112.72 (±706.54)	0.0436
FMF centile for weight	42.17 (± 34.42)	42.3 (± 29.64)	0.989
SGA/IUGR (n/N, %)	4/12 (33·33%)	5/46 (10.86%)	0.0556

^#^Not adjusted for gestational age; FMF, Fetal Medicine Foundation; SGA, Small for gestational age; IUGR, Intrauterine growth restriction.

**Figure 6:**
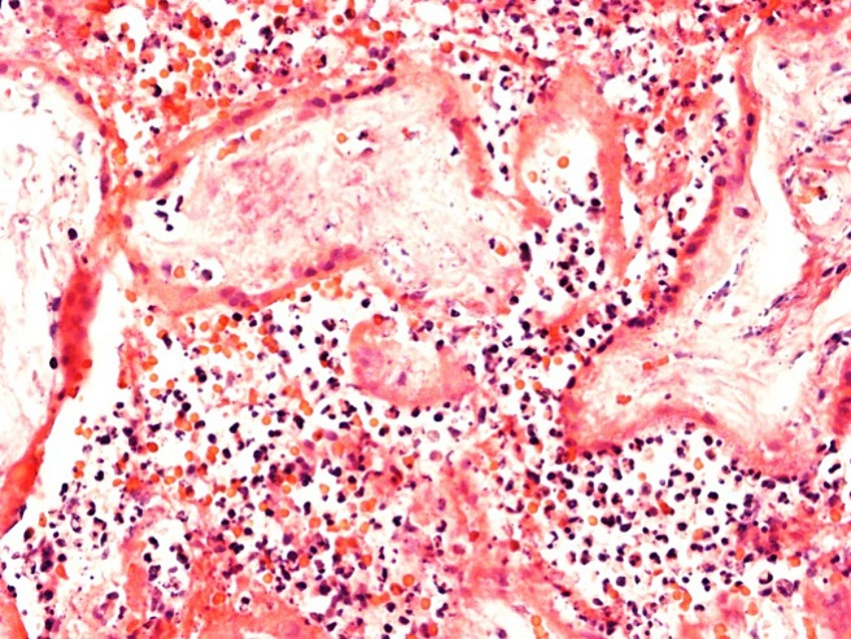
Evidence of marked chronic histiocytic intervillositis. Representative images of case 2i with stillbirth at 30 weeks of gestation.

In Case2i (stillbirth at 30 weeks of gestation), placental qPCR was positive for SARS-CoV-2. Pathology showed marked CHI (Table 3; Figure 6), whereas variant sequencing revealed the B.1.617 (Delta) variant. Alltogether, two of the stillbirth cases in the infection group were recruited during Delta, whereas Case3i was recruited during Omicron dominance.

qPCR from fetal membranes, placenta, and vaginal fluid was negative in all remaining cases and controls.

**Figure 7:**
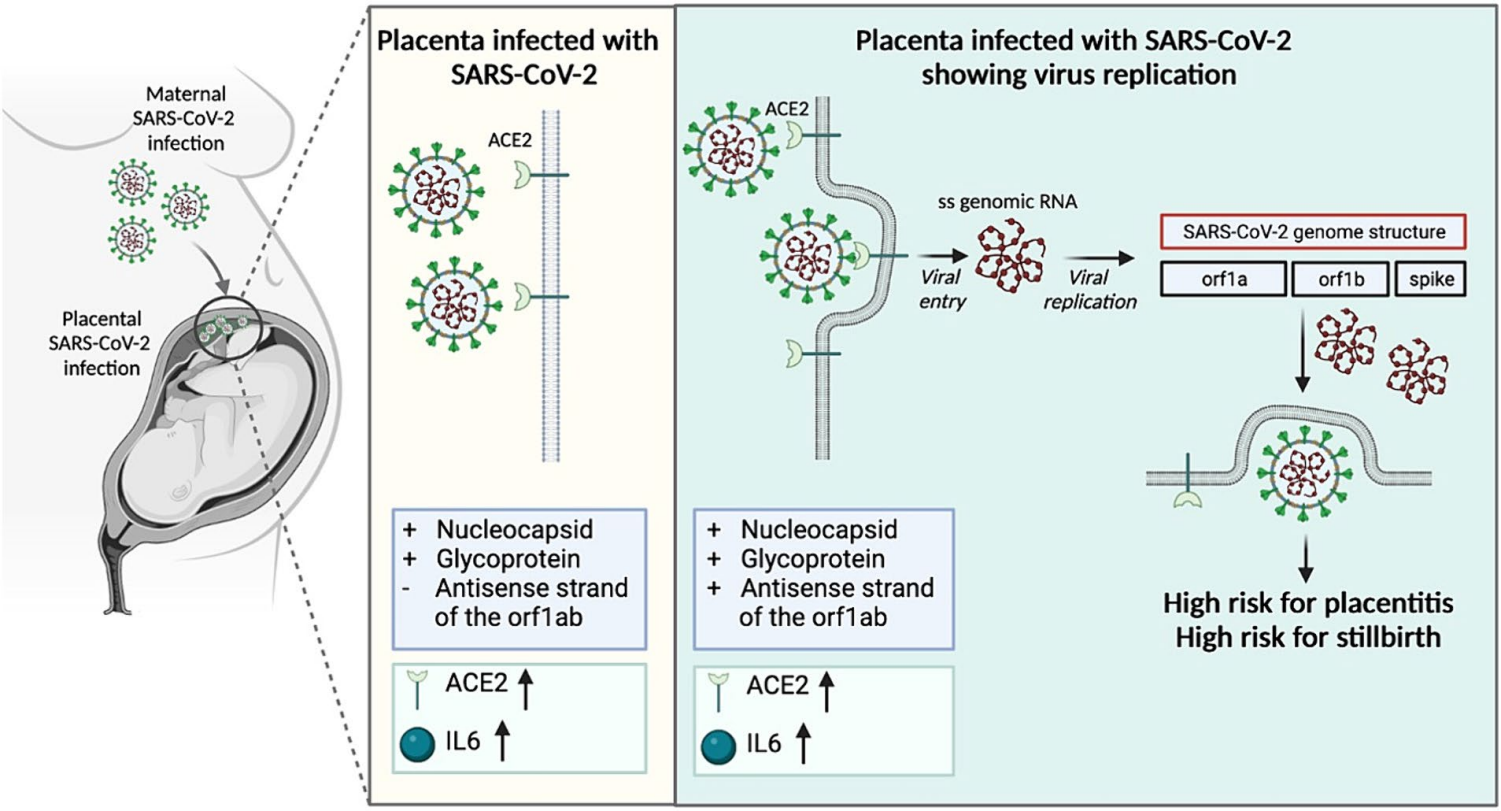
Mechanism of placental damage in SARS-CoV-2 infection during pregnancy.

Placental histopathology for Case1i (miscarriage at 18 weeks of gestation) revealed fulminant villitis and chorioamnionitis with extensive placental infarction and for Case2i massive proliferation of the intervillous fibrin with single intercalated lymphocytes and macrophages (Figures 6, 7).

## Discussion

The main result of our study is evidence of intense SARS-CoV-2 replication in 4.91%, and low to moderate replication in further 19.67% of the placentas originating from women infected with SARS-CoV-2 at any time point during pregnancy. The three patients, for whom intense replication was documented, all presented at our service with miscarriage/stillbirth, which could suggest a direct causality between infection and the intrauterine fetal demise (IUFD). Meanwhile, placental histopathological alterations in these cases reinforce this hypothesis.

Globally, intensity of placental viral replication was higher in women with severe/critical symptoms, supporting the rationale that severity of SARS-CoV-2 infection could correlate with the severity of placentitis. Furthermore, viral presence was confirmed in an important number of placentas by additional markers at protein (glycoprotein and nucleocapsid) and RNA (S gene) levels, reinforcing the results. Analog to the intensity of the replication, virus levels (IRS for glycoprotein and nucleocapsid) both correlated with the severity of maternal disease. No viral evidence could be found in the control placentas by any method, which underlines the accuracy of the results.

The susceptibility of the human placenta to viral replication was demonstrated in an advanced *ex vivo* model, with authors providing evidence that SARS-CoV-2 can undergo a full replication cycle in the placenta, which offers an equivalent for our findings (21). Our study does not only provide important data regarding the potential of SARS-CoV-2 to induce placental damage, but also on its dimensions, by quantifying this in the context of a larger pregnant population, in respect to international recommendations (3).

Concerning the timing of infection, we noted higher scores for viral replication when delivery occurred shortly after infection. Out of a clinical perspective, this suggests the existence of a `critical phase` in the first weeks after maternal SARS-CoV-2 infection. Of course, larger studies with clinical correlation are needed to support this hypothesis.

All cases with presence of intense replication occurred during the Delta dominance, underlying once again that this variant of SARS-CoV-2 was the most severe and virulent strain, characterized by its significantly higher transmissibility and increased risk of severe disease compared to earlier variants (22).

ACE2 levels were higher in SARS-CoV-2 infected placentas in our cohort, particularly in the decidua. This is in line with a recent publication describing decreasing ACE2 levels in healthy placentas thorough the pregnancy versus a persistence in case of COVID-19 infection (23). Contrastingly, Verma et al. reported a decrease in ACE2 expression in SARS-CoV-2 infected placentas, compared to controls, supporting the hypothesis of ACE2 viral colonization and thus SARS-CoV-2 associated reduction of ACE2 receptors (24). Interestingly, Roberts et al. reported a varying ACE2 expression thorough pregnancy, suggesting that early pregnancies could be more susceptible to SARS-CoV-2 infection (25). Data regarding ACE2 expression in the placenta throughout pregnancy, with or without SARS-CoV-2, remain conflicting (21). Since vertical transmission of SARS-CoV-2 only reaches 1.1–3%, a well-functioning protective mechanism seems to exist at placental level, yet this is not completely elucidated (26–28).

SARS-CoV-2 infection did not lead to SGA (small for gestational age) fetuses in our cohort. In a previous study, we could show that SARS-CoV-2 infection during pregnancy can lead to low weight placentas, also without association with IUGR (29).

Further obstetrical outcomes of our population were in line with available publications (30, 1–2).

At this point, we need to mention that controls were only matched for gestational age and obstetrical pathology, but not for maternal morbidity. This remains a limitation of our study. A further limitation is the small number of cases with IUFD, which makes it difficult to obtain robust statistics. Another important limitation is the lack of serotype analysis, which was not standardly performed in each case, in line with internal hospital practices of the respective period.

Meanwhile, major strengths of our report are its prospective nature and its case–control design. Furthermore, all staining and examinations, including IHC and ISH, have been conducted in all 71 cases, which makes our study, to our knowledge, one the most extensive of its kind. IHC and ISH were concordant in all cases, which is another strength of the study.

We report evidence of active *in vivo* SARS-CoV-2 replication in the placenta after maternal infection in pregnancy in a case–control setting in a larger population. Replication was maximal in cases of stillbirth, which suggests direct placental involvement in the pathophysiology of this dramatic outcome. In our opinion, evidence of viral replication at the maternal-fetal interface reinforces the necessity of systematically examining the placenta for SARS-CoV-2 in all stillbirth cases, even in absence of maternal COVID-19 diagnosis or infection signs. Understanding the causes of stillbirth remains essential for counseling in regards to future pregnancies.

Continuing to advocate for preventive measures against COVID-19 during pregnancy, including (re)vaccination, as well as appropriately counseling women with diagnosed infection, are of utter importance.

## Data Availability

The datasets presented in this study can be found in online repositories. The names of the repository/repositories and accession number(s) can be found in the article/Supplementary material.
